# Effects of Different Anticoagulation Doses on Moderate-to-Severe COVID-19 Pneumonia With Hypoxemia

**DOI:** 10.7759/cureus.43389

**Published:** 2023-08-12

**Authors:** Amin Ur Rehman Nadeem, Syed M Naqvi, Kurian G Chandy, Venkata V Nagineni, Rashid Nadeem, Shreya Desai

**Affiliations:** 1 Department of Critical Care Medicine, James A Lovell Federal Healthcare Center, Chicago Medical School at Rosalind Franklin University of Medicine and Science, North Chicago, USA; 2 Department of Internal Medicine, Chicago Medical School at Rosalind Franklin University of Medicine and Science, North Chicago, USA; 3 Department of Internal Medicine, Research Medical Center, Kansas, USA; 4 Department of Critical Care Medicine, Dubai Hospital, Dubai, ARE; 5 Department of Hematology and Oncology, Georgia Cancer Center at Augusta University, Georgia, USA

**Keywords:** covid 19 mortality, high-flow oxygen, non-invasive mechanical ventilation, invasive mechanical ventilation, acute hypoxemia, covid-19 pneumonia, covid 19, anticoagulation in covid-19

## Abstract

Background

COVID-19 is a prothrombotic disease that can cause thromboembolism and microthrombi, which could lead to multiorgan failure and death. Since COVID-19 is a relatively new disease, there are guidelines for anticoagulation dosing for COVID-19 patients without consensus on the dosing. We studied the effects of different doses of anticoagulation in hospitalized patients with COVID-19 pneumonia and hypoxemia on any differences in need for high-flow oxygen, mechanical ventilation, and mortality. We also analyzed the patient population who benefited most from anticoagulation.

Methodology

We performed a retrospective chart review of all patients who were admitted with the diagnosis of COVID-19 infection with positive polymerase chain reaction, pneumonia (confirmed either by chest X-ray or CT chest), and hypoxemia (oxygen saturation of <94%, while on room air). These patients were studied for outcomes (the need for high-flow oxygen, the requirement for mechanical ventilation, and overall mortality) for different doses of anticoagulation (prophylactic, escalated, and therapeutic).

Results

The sample consists of 132 subjects, predominantly males (116, 87%), with a mean age of 59 years and a standard deviation of 15. About one-third of the participants had diabetes, and more than 50% had hypertension. Additionally, 27 (20.3%) had a history of heart disease, and 70 (53%) of the subjects were admitted to the intensive care unit (ICU) at some point during the study. Among those admitted to the ICU, about 11 (8%) subjects required mechanical ventilation and 16 (12%) passed away during the study. Those who died had higher use of high-flow oxygen, noninvasive mechanical ventilation, and invasive mechanical ventilation and had a longer stay on mechanical ventilation.

There was no significant difference in mortality or need for mechanical ventilation for any strategy of anticoagulation.

Conclusions

Different doses of anticoagulation did not show any statistically significant relationship between the need for mechanical ventilation and mortality. More patients on high-flow oxygen had received escalated doses of anticoagulation as compared to those who were not on high-flow oxygen. Anticoagulation levels did not have any statistically significant effect on overall survival of patients.

## Introduction

COVID-19, caused by the SARS-CoV-2, has resulted in a global pandemic, with a significant impact on healthcare systems worldwide [1]. The United States was severely affected by COVID-19 becoming the country with the highest number of cases and deaths in 2020 [2]. COVID-19 spread exponentially from the first known case in the United States in January 2020 to one of the leading causes of death among aged above 45 years, from March to October 2020, and daily deaths frequently surpassing deaths due to heart disease during 2020 [3].

COVID-19 infection affects mainly the lungs manifesting as viral pneumonia presenting with cough, shortness of breath, and fever. COVID-19 also affects other organ systems less frequently, including gastrointestinal, renal, cardiovascular, hepatobiliary, and nervous systems [4]. Postmortem studies conducted on COVID-19 death cases reveal lungs are the most common organs affected by COVID-19 [5]. The most common finding in pathology is diffuse alveolar damage with pulmonary edema, hemorrhages, and microthrombi. The heart, liver, kidney, lymph nodes, and central nervous system are other less frequently affected organs in postmortem studies [5]. COVID-19 is a prothrombotic disease that can cause venous, arterial thromboembolism, and microthrombi. Microcirculation disorders caused by microthrombosis and acute respiratory distress syndrome caused by diffuse alveolar damage are the main causes of death in COVID-19 [6].

Multiple hypotheses were proposed for the prothrombotic state of COVID-19, including direct activation of platelets through direct interaction of virus spike protein to angiotensin-converting enzyme-2 (ACE-2) receptor on platelets [7]. Disruption of the renin-angiotensin-aldosterone system is also proposed to cause the prothrombotic state of COVID-19. COVID-19 infection is proposed to downregulation of ACE-2 leading to an increase in angiotensin II (Ang II), which has a pro-oxidative and proinflammatory effect, and Ang II increases the production of plasminogen activator inhibitor-I (PAI-I), which inhibits fibrinolysis [8]. Cytokine storm due to activation of macrophages and hyperinflammation due to defective viral clearance leads to the production of proinflammatory cytokines like interleukin-6 (IL-6), tumor necrosis factor-alpha (TNF-α), IL-17a, and formation of neutrophil extracellular traps causing endothelial damage and microthrombi formations [9,10].

Since thromboembolism is thought to be one of the major causes of mortality in COVID-19 patients, multiple studies were done to study the role of anticoagulation in COVID-19 patients. We aimed to study the effects of different doses of anticoagulation in all the hospitalized COVID-19 patients at our healthcare center with pneumonia and hypoxemia to see if there are any differences in the need for high-flow oxygen, the requirement for mechanical ventilation, and overall mortality.

## Materials and methods

We performed a retrospective chart review of all patients who were admitted with a diagnosis of COVID-19 pneumonia (confirmed either by chest X-ray or CT of the chest) and hypoxemia (defined as oxygen saturation of <94% while breathing room air) from March 1, 2020, to February 28, 2022, at Captain James A. Lovell Federal Health Care Center. Those patients had confirmed COVID-19 by performing polymerase chain reaction (PCR) on nasopharyngeal swabs or similar tests of equivalent sensitivity and specificity to detect SARS-CoV-2.

There were a total of 132 patients who met the inclusion criteria for our study. Demographics and relevant comorbidities were collected from the day of admission. The study excluded patients who had COVID-19 but were asymptomatic, as well as those without pneumonia and hypoxemia. Additionally, individuals who were not on anticoagulation during their hospital stay, those who had care withdrawn, were actively dying, or receiving terminal or hospice care, and those with a history of COVID on a prior admission were also excluded from the study. All subjects included in the study were categorized into three anticoagulation groups prophylactic, escalated, and therapeutic. These patients were studied for outcomes (the need for high-flow oxygen, the requirement for mechanical ventilation, and overall mortality) for different doses of anticoagulation administered during the first week of admission. Escalated dose of anticoagulation was defined as a high prophylactic dose that was lower than the therapeutic dose for the given agent.

Statistical analysis

Statistical analysis was performed by the IBM SPSS Statistical program (Version 22, IBM Corp., Pittsburgh, PA, USA). Statistical analysis consisted of the computation of means and standard deviations (SDs) for continuous variables and frequencies for categorical variables. Differences in continuous variables between the two study groups were analyzed by independent sample t-test. Differences in categorical variables were analyzed using the chi-square test or Fisher’s exact test as appropriate. A two-sided alpha error of *P* ≤ 0.05 was considered statistically significant.

## Results

The sample includes 132 subjects with a mean age of 59 years and an SD of 15. The majority of the sample was males (116, 87%), 43 (38%) had diabetes, and about 71 (60%) had hypertension. About 27 (20%) had a history of heart disease. Half of them were admitted to ICU at some point, with about 11 (8%) requiring mechanical ventilation (Table 1). Sixteen (12%) subjects died. The composition of individuals who died versus those who survived for the collected variables is presented in Table 2. Those who died had higher use of high-flow oxygen, noninvasive mechanical ventilation, and invasive mechanical ventilation and had a longer stay on mechanical ventilation.

**Table 1:** Sample characteristics. BMI, body mass index; NIV, noninvasive ventilation; BIPAP, bilevel positive airway pressure

Variable	*n* (%)	Mean (SD)
Age (years)	132 (100)	63.42 (19.03)
Gender (male)	116 (87.8)	
Gender (female)	16 (12.12)	
Diabetes	51 (38.3 )	
BMI (kg/m^2^)		30.4 (19.03)
Hypertension	81 (60.9)	
Coronary disease	27 (20.3)	
Renal failure	12 (9 )	
Dialysis	3 (2.3)	
ICU admission	70 (53)	
Anticoagulation		
Prophylactic	84 (63.2)	
Escalated	29 (21.8)	
Therapeutic	19 (14.3)	
Hypoxemia	132 (99)	
High-flow oxygen use	47 (35.3)	
NIV (BIPAP use)	17 (12.8)	
Mechanical ventilation	11 (8.3)	
Remdesivir	96 (72.2)	
Steroids	95 (71.4)	
Baricitinib	15 (11.3)	
Antibiotics	104 (78.2)	
Tocilizumab	5 (3.8)	
Hydroxychloroquine	8 (6)	
Plasma therapy	7 (5.3)	
New organ failure	37 (27.8)	
Days on a ventilator		1.5 (8.6)
Died	16 (12.1)	

**Table 2:** Comparison of patients who survived versus died. ^*^A two-sided alpha error of *P* ≤ 0.05 was statistically significant. BIPAP, bilevel positive airway pressure; BMI, body mass index; SD, standard deviation

Variable	Survived (116, 97.8%)		Died (16, 12.1%)		P-value
	n (%)	Mean (SD)	n (%)	Mean (SD)	
Gender (Male)	102 (88)		14 (88)		0.96
Gender (Female)	14 (12)		2 (12)		0.96
Diabetes	43 (37)		8 (50)		0.41
Hypertension	71 (61.2)		10 (44.3)		0.92
Coronary heart disease	25 (21.5)		2 (12.5)		0.73
Renal failure	9 (7.7)		3 (18.7)		0.15
Dialysis	2 (1.7)		1 (16.6)		0.32
New organ Failure	29 (25)		8 (50)		0.07
Remdesivir	84 (72.4)		12 (75)		1.0
Steroids use	84 (72.4)		11 (68.7)		0.76
Baricitinib	10 (8.6)		5 (31.2)		0.02^*^
Antibiotics	88 (75.8)		16 (100)		0.02^*^
Tocilizumab	4 (3.4)		1 (6.2)		0.48
Hydroxychloroquine	7 (6)		1 (6.2)		0.97
Plasma therapy	4 (3.4)		3 (18.7)		0.03^*^
Hypoxemia	116 (100)		16 (100)		0.81
High-flow oxygen use	35 (30.1)		12 (75)		0.01^*^
BIPAP use	8 (6.8)		10 (62.5)		0.01^*^
Ventilator use	5 (4.3)		6 (37.5)		0.01^*^
Age		61.9 (19.3)		74 (12.7)	0.55
BMI (kg/m^2^)		30.6 (6.6)		29.4 (10.2)	0.09
Days on ventilator		0.82 (6.25)		6.44 (17.8)	0.01^*^

There was no significant difference in mortality (Table 3) or the need for mechanical ventilation (Table 4) among patients who received prophylactic, escalated, or therapeutic anticoagulation. These results are not reliably significant owing to a small number of subjects.

**Table 3:** Mortality among three groups of AC. AC, anticoagulation

AC group	Survived, *n* (%)	Died, *n* (%)	*P*-value
Preventive	75 (64.6)	9 (56.2)	0.63
Escalated	24 (20.6)	5 (31.2)	0.65
Therapeutic	17 (14.6)	2 (12.5)	0.75

**Table 4:** Comparison of the need for mechanical ventilation among the three groups of AC. AC, anticoagulation

AC group	Intubated (*N* = 11), *n* (%)	Not intubated (*N* = 121), *n* (%)	*P*-value
Preventive	6 (54.5)	78 (64.4)	0.46
Escalated	4 (36.3)	25 (20.6)	0.49
Therapeutic	1 (9)	18 (14.8)	0.85

## Discussion

In this single-center retrospective chart review study, different doses of anticoagulation did not show any statistically significant relationship between the need for mechanical ventilation and mortality. More patients on high-flow oxygen had received escalated doses of anticoagulation as compared to those who were not on high-flow oxygen. Our study analyzes the effects of different doses of anticoagulation on patients with COVID-19 infection with confirmed pneumonia and hypoxemia. SARS-CoV-2 (COVID-19) infection is a global pandemic that has resulted in more than 768 million cases worldwide [1]. It has impacted nearly every country across the globe. In earlier studies, prophylactic anticoagulation in patients with high D-dimer levels was shown to have better survival [11]. Paranjpe et al. reported that therapeutic full-dose anticoagulation in critically ill patients led to better survival [12]. Tacquard et al. showed that high-dose prophylactic anticoagulation is associated with a reduction in thrombotic complications in critically ill patients with COVID-19 without an increased risk of hemorrhage [13]. In their study, Al-Samkari et al. demonstrated that among critically ill adults with COVID-19, early therapeutic anticoagulation did not have a significant impact on survival in the target trial emulation [14]. Sadeghipour et al. reported intermediate dosing of anticoagulation is not associated with better survival [15]. Most of the studies involving the effects of anticoagulation on COVID-19 infection primarily analyzed thrombotic and bleeding complications [13,14]. The issue of what is the right dose of anticoagulation is far from resolved; studies have shown different doses with favorable versus no effects [12-15].

In our study, we analyzed the effects of different doses of anticoagulation on patients with COVID-19 pneumonia and hypoxemia for the use of high-flow oxygen, mechanical ventilation, and hospital mortality. Multiple hypotheses have been proposed regarding the prothrombotic state of COVID-19. These hypotheses include the direct activation of platelets through the virus spike protein's interaction with the ACE-II receptor on platelets, downregulation of ACE-II resulting in increased production of PAI-I, which inhibits fibrinolysis, and the cytokine storm-mediated increase in thrombosis [7-10]. It has also been suggested that micro-thrombosis of several vascular beds could lead to multiple organ failure and one of the major causes of death with SARS-CoV-2 infection [16-20]. What is not known is whether early use of anticoagulation at a higher than prophylactic dose could reduce mortality by inhibiting micro-thrombosis. To address the issue of anticoagulation dosing for COVID-19-related acute illness requiring hospitalization, the National Institute of Health (NIH) recommends the use of a therapeutic dose of heparin (preferably low-molecular-weight heparin) for patients with D-dimer levels above the upper limit of normal, who are not at the high risk of bleeding, are not critically ill and require low-flow oxygen. For adults who are critically ill and require intensive care unit-level care, including those who require high-flow oxygen, the NIH panel recommends using a prophylactic dose of heparin for venous thromboembolic (VTE) prophylaxis, unless it is contraindicated [21]. Similarly, the American Society of Hematology (ASH) panel issued a conditional recommendation in favor of therapeutic dose over prophylactic dose anticoagulation in patients with COVID-19-related acute illnesses who do not have suspected or confirmed VTE. The panel emphasized the need for an individualized assessment of the risk of thrombosis and bleeding. The panel recommended the use of either low-molecular-weight or unfractionated heparin. However, it is essential to note that this recommendation was based on very low certainty in the evidence, suggesting the need for randomized controlled trials to assess the effects of different doses of anticoagulation on COVID-19-related acute illness [22].

The review of patients who were admitted at our healthcare institution with COVID-19 pneumonia with hypoxemia shows that all of them received anticoagulation unless there were contraindications. We also noticed that predominantly, these patients received prophylactic doses of anticoagulation. However, patients on high-flow oxygen therapy mostly received escalated doses of anticoagulation. Our study hypothesized that escalated or therapeutic doses of anticoagulation will have favorable outcomes in patients presenting with COVID-19 pneumonia and hypoxemia. Although our study did not reveal any significant differences in the need for high-flow oxygen, mechanical ventilation, and mortality among the three anticoagulation groups, we believe this lack of significant findings is more likely due to the small sample size and the retrospective nature of the study. Based on the available literature and guidelines, anticoagulation has been recommended for COVID-19 patients who require hospitalization, as opposed to receiving no anticoagulation [12-14,21-22]. Randomized controlled trials comparing prophylactic and higher doses of anticoagulants are needed to confirm these results further. 

## Conclusions

Our study did not show any statistically significant relationship between the need for high-flow oxygen, mechanical ventilation, and mortality among three different anticoagulation dose groups. More patients on high-flow oxygen had received escalated doses of anticoagulation as compared to those who were not on high-flow oxygen. Anticoagulation doses did not show any statistically significant effect on the overall survival of patients. However, it's important to note that the limited number of patients analyzed in this study warrants a larger sample size for improved statistical power in identifying potential relationships.
